# Tumor-microenvironment and molecular biology of classic Hodgkin lymphoma in children, adolescents, and young adults

**DOI:** 10.3389/fonc.2025.1515250

**Published:** 2025-05-01

**Authors:** Tomohiro Aoki, Kyle Wierzbicki, Suhong Sun, Christian Steidl, Lisa Giulino-Roth

**Affiliations:** ^1^ Princess Margaret Cancer Centre, University Health Network, Toronto, ON, Canada; ^2^ Department of Medicine, University of Toronto, Toronto, ON, United States; ^3^ Department of Pediatrics, Weill Cornell Medicine, New York, NY, United States; ^4^ Centre for Lymphoid Cancer, British Columbia Cancer, Vancouver, BC, Canada; ^5^ Department of Pathology and Laboratory Medicine, University of British Columbia, Vancouver, BC, Canada

**Keywords:** Hodgkin lymphoma (HL), pediatric, TME (tumor microenvironment), genetics, Hodgkin and Reed-Sternberg cells

## Abstract

Classic Hodgkin lymphoma (cHL) exhibits a bimodal age distribution with incidence peaks in adolescents and young adults (AYAs) aged 15-39 years and in older adults over 50 years. The unique biology of cHL, characterized by a tumor microenvironment (TME) composed predominantly of non-malignant immune and stromal cells, plays a pivotal role in supporting Hodgkin and Reed-Sternberg (HRS) cells, the malignant cells of cHL. Understanding the role of the TME in cHL and its age-related differences is crucial for deciphering differential disease etiologies and developing biomarker-driven targeted therapies. Recent technical advances in single-cell sequencing and multiplexed spatial imaging have revealed age-related differences in TME composition and function, including key cellular interactions, leading to the development of age-specific prognostic indicators. In addition, advances in our ability to isolate nucleic acids from HRS cells have accelerated our understanding of the molecular alterations in cHL, many of which drive interactions within the TME. Molecular differences in cHL between pediatric/AYA and older adult patients have also emerged. This review summarizes the unique biology of cHL and its TME in children, adolescents, and young adults, highlighting recent breakthroughs in our understanding of cHL biology, differences across the age spectrum, and advances in biomarker development.

## Introduction

Classic Hodgkin lymphoma (cHL) is an aggressive B-cell malignancy that primarily affects adolescents and young adults (AYAs) aged 15-39 years (1). Many patients with cHL can achieve a cure through multi-agent chemotherapy, radiotherapy, and more recently, immunotherapies (2–9). Despite recent advances, up to 25% of patients will experience relapse or progressive disease (1, 10). In addition, chemotherapy and radiation are associated with an increased risk for late toxicities and overall morbidity. Understanding the biology of cHL and its tumor microenvironment (TME) will be key to developing novel treatment strategies as well as biomarkers to tailor therapy. cHL is unique in that the majority of the cellular tumor mass is composed of non-neoplastic immune and stromal cells which establish an extensive supportive network around the rare malignant Hodgkin and Reed-Sternberg (HRS) cells (11–13). The importance of the TME in HRS cell survival is evidenced by the success of recent therapies targeting the TME, including immune checkpoint inhibitors targeting programmed cell death protein 1 (PD-1) (3, 7, 14). Recent advances in single cell sequencing and spatial molecular imaging have allowed for an unprecedented characterization of the cHL TME. In addition, genomic characterization of cHL, which has been challenging due to the rarity of the HRS cells within a dense TME, is now possible (15–19). Collectively, this work has provided insights into the interaction between HRS cells and the TME as well as increasing evidence that the biology of cHL in pediatric and AYA patients has distinct features compared to older adults. This review will focus on the unique biology of cHL in children, and AYAs including TME and molecular features, aimed at highlighting recent breakthroughs in cHL biology and the discovery of potential biomarkers.

## Epidemiology of cHL in children and AYAs

cHL is classified into four histological subtypes based on morphology and immunophenotype: nodular sclerosis (NS), mixed cellularity (MC), lymphocyte rich (LR), and lymphocyte depleted (LD) (20). cHL exhibits a bimodal age distribution, with peaks in AYAs and older adults (over 50 years). The majority of cHL cases among AYAs are the NS subtype which is seen in approximately 76% of cases. The MC subtype is more common in younger children <10y of age where it represents 22% of cases (vs. 9% in AYAs) (21, 22).

Approximately 30% of cHL is associated with EBV (6). EBV-associated cHL is more common in patients <10y of age and those >50y (23). The MC histologic subtype is associated with EBV independent of age (23, 24). In EBV+ HL the virus exists in a latent state where it expresses EBV nuclear antigen 1 (EBNA1), latent membrane protein 1 (LMP1), and LMP2a. LMP1 is considered an oncogene which can mimic the signaling domain of CD40 and activate the NF-kB signaling patway (25). In adults age ≥45y EBV+ HL is correlated with inferior outcome compared to EBV- HL (24). The impact of EBV on outcome in pediatric and AYA HL is less clear, with some studies finding EBV associated with favorable survival, particularly in patients age <15y, some studies finding EBV associated with inferior outcome in subgroups such as those with NS histology or advanced stage, and other studies finding no association (24, 26, 27).

## Tumor-microenvironment in cHL

**Figure 1:**
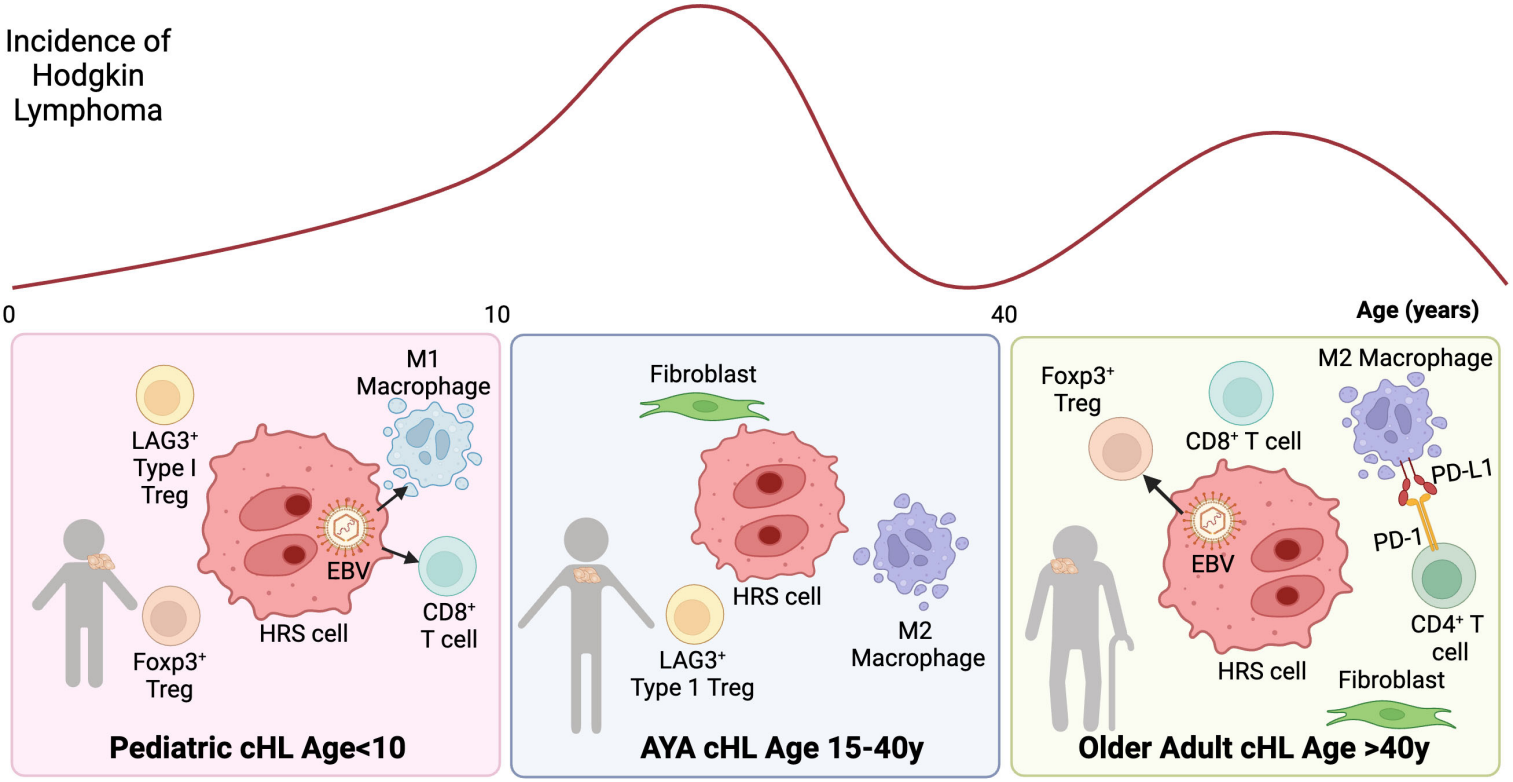
Tumor microenvironment ecosystem of cHL according to age groups. cHL presents a bimodal age distribution, with distinct TME characteristics across age groups: Left: cHL age in pediatric patients age <10y: The TME is enriched with M1 macrophages, accompanied by a diverse population of CD4 and CD8 T cells. EBV is more common in this age group and associated with M1 macrophages and cytotoxic T-cells. Center: cHL in AYAs age 15-40y: The TME is associated with M2 macrophages. A subset of AYA HL cases also exhibits LAG3+ type 1 regulatory T cells. Right: cHL in elderly: EBV positivity in HRS cells is more frequently observed in elderly patients and associated with FoxP3 regulatory T-cells. Additionally, the TME is characterized by PD-L1+ macrophages, which are often in close proximity to PD1+ CD4 T cells, indicating a potential immunosuppressive interaction.

cHL features a quantitatively dominant TME (~99%) composed of stromal cells and non-malignant immune cells, including T cells, B cells, eosinophils, and macrophages. Immune cells in the TME are educated by signals from HRS cells (20, 28, 29), including cytokines/chemokines and altered expression of cell surface molecules (30). These changes are in part linked to genetic alterations that contribute to the development of lymphoma-specific cellular ecosystems that allow escape of the malignant cells from the host immune system. The most abundant cells in the TME of cHL are CD4+T cells including helper T cells and regulatory T-cells (Tregs), which form immunosuppressive niches (31). Additionally, unique tumor-associated macrophage (TAM) subsets are present in the TME of cHL, and their presence is correlated with outcome in adults (28, 32–34). Recent studies have uncovered key differences in the cHL TME across age groups including: 1) an enrichment in M1 macrophages, and cytotoxic T-cells in younger patients age <10y, 2) increased M2 macrophages and LAG3+ type 1 regulatory T cells and AYAs age 15-39, and 3) increased FoxP3 regulatory T-cells and PD-L1+ macrophages among elderly patients (35) (**Figure 1**).

### Epstein-Barr virus (EBV) infection and its impact on the cHL TME

Key differences exist in the TME of EBV+ vs. EBV- pediatric cHL, including increased M1 macrophage polarization and a cytotoxic/Th1 viral response in EBV+ cases (35–37). In addition there are contrasts between the TME in pediatric and adult EBV+ cHL, suggesting that adults may have reduced anti-cancer immunity and an “aged” TME, which could explain the inferior clinical outcome (31, 35, 38). EBV+ cHL in adults is enriched for FOXP3+ Tregs (37, 39) and immunosuppressive cytokines such as IL-4, IL-5, IL-6, IL-9, IL-10, IL-13, and TGF-β. In contrast, in EBV+ cHL in children has increased cytotoxic/T-helper cell 1(Th1) CD8+ T-cell infiltration characterized by TIA-1 and T-bet expression (36) and M1-polarized TAMs which may potentially contribute to the favorable outcome through effective immune surveillance (31, 34). The biology underlying differences in EBV+ TME across ages including the potential role of senescence in older adults remain unclear, requiring further study across all age groups.

### Gene expression profiling of the cHL TME in children and adults

Gene expression profiling (GEP) of cHL biopsies has expanded our understanding of the TME and allowed for the development TME-based biomarkers. Scott et al. developed a 23-gene expression prognostic model, HL27 (40), which predicted outcome among adults with advanced-stage cHL. To evaluate the applicability of this model in pediatric HL, Johnston et al. performed GEP on cHL tumors from pediatric patients treated on the Children’s Oncology Group (COG) trial, AHOD0031 (5) which evaluated a risk adapted-approach to the treatment of intermediate-risk cHL. HL27 (40) did not predict outcome in the pediatric cohort, suggesting potential age-related biological differences in the TME. Indeed, Spearman correlation analysis of GEP-based TME component scores with age revealed that eosinophil, B-cell, and mast cell signatures were more prevalent in younger patients, while macrophage and stromal signatures were more pronounced in older patients (41). Based on these observations, Johnston et al. developed a distinct GEP-based predictive model for pediatric cHL, PHL-9C, which is predictive of 5-yr event-free survival (EFS) among children treated on COG AHOD0031 (41). This model is composed of: Tregs, mast cells, T helper 2 (Th2) cells, myeloid-derived suppressor cells, and HRS cells (41). We are currently investigating the utility of PHL-9C in brentuximab vedotin-containing treatment (5).

### Evaluation of the TME in cHL in adults using single cell approaches

Recent technical advances in multiparameter imaging (MPI) have allowed for a more comprehensive characterization of the TME at single cell resolution (11). These approaches provide details on co-expression patterns, cellular composition, and cell-to-cell spatial interactions, which have allowed for detailed descriptions of the architecture of the TME in cHL. A series of studies in adult cHL have utilized MPI to better understand the mechanisms of response to therapy and identify biomarkers. MPI of cHL tumors has identified a spatial relationship between TAMs that express PD-1 ligand (PD-L1) and HRS cells (42). Unexpectedly, the PD-L1+ macrophages outnumbered PD-L1+ HRS cells in the TME of cHL and co-localized with T-cells, suggesting that this interaction may be a key target of PD-1 blockade. This may explain the efficacy of PD-1 therapy in cHL despite frequent loss of B2M leading to decreased expression of MHC-I (7, 14, 43–46). Further work by Aoki et al. evaluating the TME in paired diagnostic and relapsed cHL samples identified a spatial interaction between CXCR5+ HRS cells and CXCL13+ TAMs (47). Although previous biomarker studies in cHL had revealed an association between the abundance of macrophages and clinical outcome in adult cHL (48–50), the specific cellular interactions and their relevance in refractory disease were not known. Using spatial TME information, Aoki et al. developed a model predictive of outcome in the relapsed setting (51). The model (RHL4S) is composed of spatial scores from 4 variables (51, 52), each of which is independently associated with failure free survival after autologous stem cell transplant: CXCR5+ HRS cells, PD1+ CD4+ T-cells, CD68+ macrophages, and CXCR5+ non-malignant B-cells.

Single cell suspensions of lymphoma tissue represent another resource to investigate complex TME ecosystems with high resolution (11). Utilizing time-of-flight cytometry (CyTOF), Cedar et al. found that cHL has a TME that is enriched for CD4+ T-cells. Within this population there is an expansion of Th1 polarized CD4+ T effector cells that express PD-1, and may represent another relevant target of PD-1 blockade. This work also identified Th1 polarized Tregs which did not express PD-1 (53). More recently, single cell RNA sequencing (scRNA-seq) has provided an opportunity to define the phenotypes of individual HRS cells and immune cells in the cHL TME (51, 54, 55). Aoki et al. integrated both scRNA-seq and MPI to study cHL in adults. This work has defined an interaction between HRS cells and type 1 regulatory (Tr1) T-cells with enhanced expression of the inhibitory receptor LAG3. LAG3+ T-cells, which are functionally immunosuppressive, co-localize with HRS cells that do not express MHC class II (54), providing a potential mechanism for immune evasion in this cellular subset.

### Evaluation of the TME in pediatric cHL using single cell approaches

To date, single cell level studies in cHL have largely been restricted to adult cohorts. There is evidence, however, that the TME at the single cell level may differ across age groups. In a recent study Stewart et al. performed scRNA-seq and MPI on cHL cases and integrated their data with an earlier report (56) to include cases across the age spectrum (6-80y). This work identified an abundance of mononuclear phagocytes including dendritic cells and monocytes in the vicinity of HRS cell and found that these cells express immune checkpoints including PD-L1 and TIM3. The expression of immune checkpoints in mononuclear phagocyte populations increased with age (56), indicating the potential for distinct myeloid cell biology across age groups. Indeed, as opposed to the strong evidence in adult cHL (50) for a prognostic role of TAMs, this relationship is not observed in pediatric cHL (31, 35, 57).

Other studies evaluating the TME in pediatric cHL on the single cell level have been limited to flow cytometry and traditional imaging techniques. In a study evaluating the TME in pediatric cHL by flow cytometry, cHL cases were found to have high CD7 expression and an expansion of CD45RO+ T-cells. Of note, elevated CD7 has also been described in adult cHL (58, 59). An investigation of LAG3+ T-cells in pediatric cHL using IHC on patients treated on the COG AHOD0031 revealed that 73/115 (63%) of the baseline pediatric cHL tumors demonstrated LAG3+ staining in the TME (60).

## Genomic alterations in HRS cells

Genomic characterization of cHL has previously been limited by the low abundance of HRS cells, often leading to <1% of the nucleic acids being derived from malignant cells in bulk sequencing assays. This has been overcome with two complementary approaches: the first is to purify HRS cells using either laser capture microdissection or fluorescence activated cell sorting (FACS); the second is to profile the genomic composition of HRS cells using circulating tumor DNA (ctDNA), which is abundant in relatively high quantities in cHL despite the rarity of HRS cells (8, 61, 62). Collectively these advances have allowed for whole genome, whole exome, and targeted sequencing of cHL in large cohorts across the age spectrum (15, 16, 19, 63–66). This work revealed a genomically complex tumor with high ploidy including whole genome duplications, complex structural variants, and a high mutational burden (19). cHL cases harbor frequent alterations in genes involved in immune evasion and dysregulation of JAK/STAT and NF-κB signaling. Key differences in the cHL molecular profile have emerged across age groups. In the following sections, we will focus on recently identified genomic alterations in cHL that advance our understanding of the interaction between HRS cells and the TME as well as distinct features of the cHL genome in pediatric/AYA patients.

### Genomic mechanisms of immune evasion

**Figure 2:**
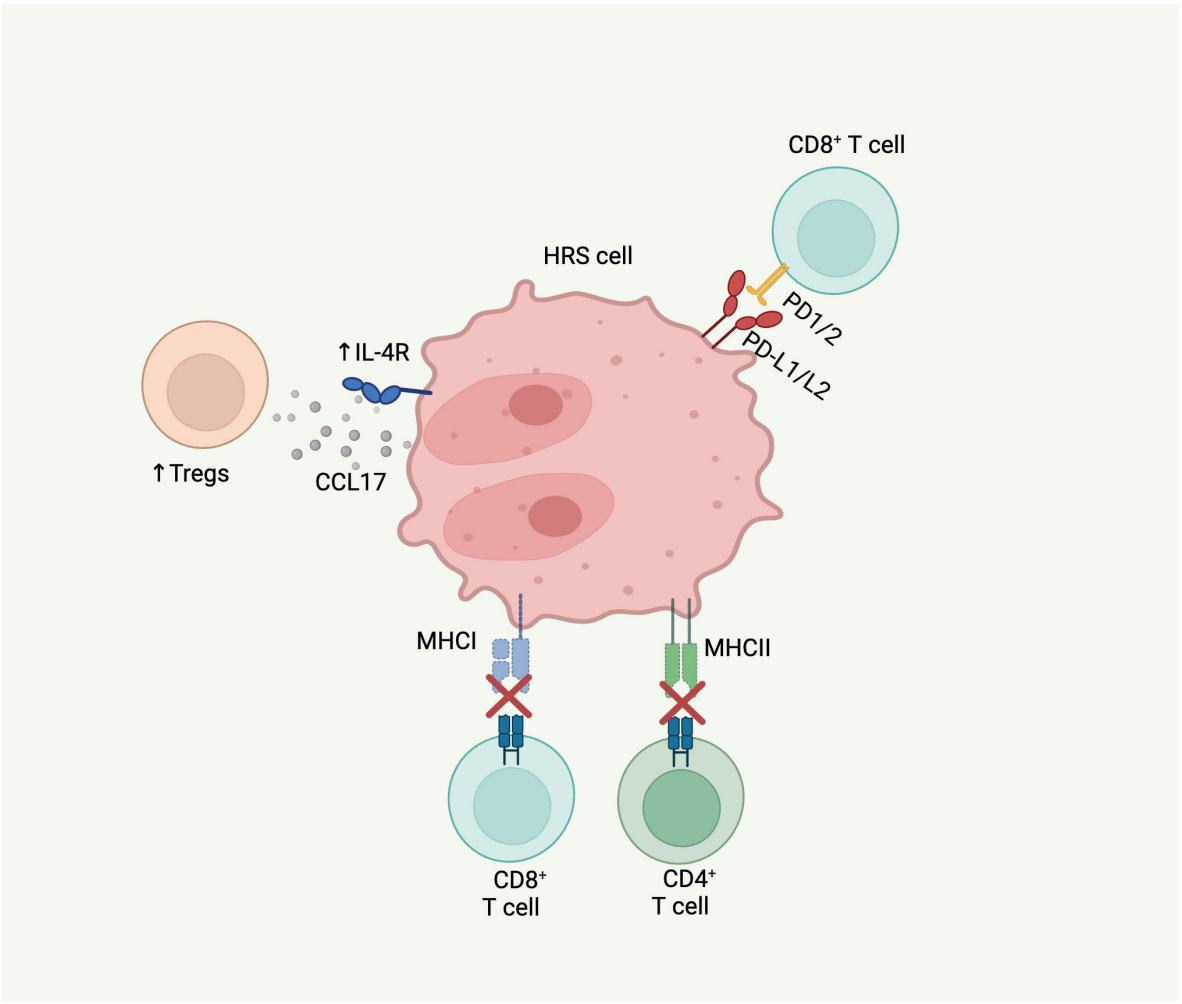
Molecular alterations in cHL that contribute to TME. HRS cells can modulate the immune microenvironment to evade tumor clearance by immune cells through several mechanisms: 1) upregulation of PD-L1/L2 leading to inhibition of CD8 T cell activation and proliferation; 2) *B2M* alterations resulting in MHC class I complex impairment and subsequent immune evasion by CD8 T cells; 3) *CIITA* alterations leading to MHC class II complex downregulation with subsequent immune evasion by CD4 T cells; and 4) *IL-4R* gain-of-function mutations causing increased downstream synthesis and release of CCL17, a regulatory T cell attractant.

HRS cells harbor several genomic alterations that enable them to modulate the TME resulting in immune privilege (**Figure 2**). Upregulation of programmed death-ligand 1 (CD274/PD-L1) and 2 (PDCD1LG2/PD-L2) is a hallmark of HRS cells and a key mechanism of immune evasion (67). Overexpression of PD-L1 and PD-L2 can occur via polyploidy/genome duplication, arm-level gain of 9p, or focal amplification of 9p24.1, leading to amplification of both the PD-L1 and PD-L2 gene loci (18, 68). 9p24.1 amplification is detected in approximately 75% of cases (18, 69). Immune evasion by HRS cells is further mediated by alterations in the MHC class I and II, which decrease the recognition of malignant cells by cytotoxic CD8+ and CD4+ T cells, respectively.

Genomic sequencing of HRS cells and ctDNA from patients with cHL have identified loss-of-function alterations in B2M, a subunit on MHC class I, in 33-70% of cHL cases (15, 18, 19). In most cases, *B2M* alterations are biallelic. Ectopic expression of *B2M* in a HL cell line with *B2M* mutation restored MHC I expression, highlighting the key role of these alterations in loss of MHC I in cHL (15). In an examination of B2M protein expression in a large cohort of cHL tumors by immunohistochemistry, it was observed that B2M loss was more common in younger patients (median age 30 vs. 47, p<0.0001), suggesting that this mutation may be a feature of cHL in children and AYAs (15). Alterations in other components of MHC-I have also been described in HRS cells. Truncating and missense mutations in *HLA-B* have been identified in 17-20% of cHL cases (18, 19). Additional structural variants and complex events involving *HLA-B* have also been reported (19).

Alterations impacting MHC class II also occur recurrently in cHL. Structural variants in Class II Major Histocompatibility Complex Transactivator (*CIITA*), which regulates transcription of MHC class II genes, are observed in 9-16% of cases (19, 70, 71). Structural variants, including balanced translocations, and complex rearrangements of *CIITA* downregulate *CIITA* and are associated with reduced expression of MHC class II (18, 70, 72).

Gain-of-function mutations in interleukin-4 receptor (IL-4R), which mediates IL-4 and IL-13 signaling, are present in 5-10% of cHL cases (19, 63). The majority of these mutations are truncating and clustered at the cytosolic C terminus of the IL-4R protein, leading to disruption of the immunoreceptor tyrosine-based inhibitory motif (ITIM). *In vitro* studies have demonstrated that *IL-4R* mutations induce downstream expression of STAT6, leading to increased synthesis of CCL17, a regulatory T cell attractant, providing another possible mechanism of immune tolerance (63).

### Genomic subtypes of cHL and differences across age groups

As information emerges regarding the genomic landscape of cHL, key questions that can now be addressed include: 1) can cHL be divided into clinically relevant molecular subtypes?; 2) is cHL in pediatrics distinct from that in adults? In a recent report of whole genome sequencing of HRS cells from pediatric and adult cases, a higher mutational burden was observed in pediatric and AYA patients compared to older adults >50y. In addition, an accelerated rate of the “aging” mutational signatures SBS1 and SBS5 was observed among pediatric and AYA patients (19). The increased mutational burden was independent of sequencing coverage and EBV status. This suggests that pediatric and AYA cases may have a distinctively high mutational burden which is not observed in older adults.

Molecular subtypes of cHL have been defined from a large cohort of cases (n=366) that were profiled using ctDNA in a recent report by Alig et al (58). In this work, two clusters emerged: the H1 cluster is characterized by younger age (median age = 30y vs. 42y in the H2 cluster, p=0.02), higher mutational burden, and mutations in NF-kB, JAK/STAT and PI3K signaling. The H2 cluster had a more even age distribution and was defined by lower mutational burden, more frequent somatic copy number alterations, and mutations in TP53 and KMT2D. Patients in the H2 cluster had an inferior progression free survival. In addition, an analysis of the transcriptional signature of patients >65y revealed a distinct signature defined by lower rates of cytokine response over T-cell activation signatures. Heger et al. also recently studied a cohort of 243 patients with cHL by both ctDNA to profile HRS cells and RNAseq to characterize the TME (73). This work identified three clusters of HL: “oncogene driven HL”, which resembles the H1 cluster identified by Alig et al. with high mutational burden, alterations in HL driver genes, and a cold TME; “inflammatory immune escape HL” characterized by copy number changes resulting in immune escape; and “virally-driven HL” which was enriched in cases with EBV and/or human herpes virus 6. Aoki et al. independently identified 4 molecular subgroups using DNA of enriched HRS cells, which revealed cluster correlations with clinical parameters (e.g. EBV status and age) similar to those described in Alig et al. (H1/H2 clusters) (74, 75). Additionally, each of the four molecular subgroups demonstrated distinctive TME correlates and unique gene expression signatures in HRS cells.

Despite differences in methodology used to define molecular subtypes—including sequencing methods, clustering algorithms, and the number of genes analyzed—all molecular classifications have identified certain common subtypes associated with mutational burden, age, and EBV status. However, the underlying biology of each molecular subtype and their targetable vulnerabilities remain unknown. Moving forward, further biological investigations and methodological optimizations, including the development of publicly available tools applicable to individual cases such as the LymphGen system, will be essential to implement the classification system in future clinical trials and/or routine clinical practice (76).

## Conclusion

Recent technological advances have provide us with new opportunities to study the biology of cHL and its TME at an unprecedented resolution (77). Although pediatric cases have been included in some series, additional research specifically focusing on pediatric cHL is needed. There is increasing evidence suggesting that the biology of cHL may differ between pediatric/AYA and older adult patients, in both the TME composition and the genomic profile of HRS cells. Therefore, further collaborative efforts between pediatric and adult groups are critical next steps to understand the disease biology across all age groups. Encouragingly, recent collaborations between pediatric and adult cooperative groups have expanded the opportunity to harmonize the development of clinical trials in cHL across different age groups (22, 78). These trials will afford us the opportunity to study cHL biology across the age spectrum in uniformly treated cohorts. Through these efforts, we aim to further delineate the biology of cHL across the age spectrum, leading to risk-adapted targeted treatments that improve treatment outcomes with minimal toxicity for both children and adults with cHL.
